# The role of glucocorticoid receptor phosphorylation in Mcl-1 and NOXA gene expression

**DOI:** 10.1186/1476-4598-9-38

**Published:** 2010-02-15

**Authors:** James T Lynch, Ramkumar Rajendran, Georgia Xenaki, Ilhem Berrou, Constantinos Demonacos, Marija Krstic-Demonacos

**Affiliations:** 1 School of Pharmacy and Pharmaceutical Sciences, Stopford Building, The University of Manchester, Oxford Road, Manchester, M13 9PT, UK; 2 Faculty of Life Sciences, Michael Smith Building, The University of Manchester, Oxford Road, Manchester, M13 9PT, UK

## Abstract

**Background:**

The cyclin-dependent kinase (CDK) and mitogen-activated protein kinase (MAPK) mediated phosphorylation of glucocorticoid receptor (GR) exerts opposite effects on GR transcriptional activity and affects other posttranslational modifications within this protein. The major phosphorylation site of human GR targeted by MAPK family is the serine 226 and multiple kinase complexes phosphorylate receptor at the serine 211 residue. We hypothesize that GR posttranslational modifications are involved in the determination of the cellular fate in human lymphoblastic leukemia cells. We investigated whether UV signalling through alternative GR phosphorylation determined the cell type specificity of glucocorticoids (GCs) mediated apoptosis.

**Results:**

We have identified putative Glucocorticoid Response Elements (GREs) within the promoter regulatory regions of the Bcl-2 family members NOXA and Mcl-1 indicating that they are direct GR transcriptional targets. These genes were differentially regulated in CEM-C7-14, CEM-C1-15 and A549 cells by glucocorticoids and JNK pathway. In addition, our results revealed that the S211 phosphorylation was dominant in CEM-C7-14, whereas the opposite was the case in CEM-C1-15 where prevalence of S226 GR phosphorylation was observed. Furthermore, multiple GR isoforms with cell line specific patterns were identified in CEM-C7-14 cells compared to CEM-C1-15 and A549 cell lines with the same antibodies.

**Conclusions:**

GR phosphorylation status kinetics, and site specificity as well as isoform variability differ in CEM-C7-14, CEM-C1-15, and A549 cells. The positive or negative response to GCs induced apoptosis in these cell lines is a consequence of the variable equilibrium of NOXA and Mcl-1 gene expression potentially mediated by alternatively phosphorylated GR, as well as the balance of MAPK/CDK pathways controlling GR phosphorylation pattern. Our results provide molecular base and valuable knowledge for improving the GC based therapies of leukaemia.

## Background

Glucocorticoid hormones (GCs) are widely used for the treatment of medical conditions such as asthma and pulmonary diseases, inflammatory bowl disease, rheumatoid arthritis and Acute Lymphoblastic Leukaemia (ALL) [1-5]. The ability of GCs to suppress inflammation and induce apoptosis is the main factor contributing to their therapeutic activity.

GCs exert most of their physiological responses by binding to and modulating the transcriptional activity of the glucocorticoid receptor (GR). GR is a member of the subfamily of steroid receptors that is part of the superfamily of nuclear receptors. GR binding to the Glucocorticoid Response Elements (GREs) present in the promoters of its target genes is the mechanism by which the expression of these genes is regulated by glucocorticoids. Positive and negative GREs [6,7], protein-protein interactions between GR and its numerous co-factors [3,8-10] and with other transcription factors such as AP-1, NF-κB, CREB, and GATA-1 determine the outcome of the GR mediated regulation of gene expression [2,4,6,9]. Posttranslational modifications of GR are another way of regulation of its target gene specificity and involve several cell-signalling cascades [10].

Phosphorylation sites have been identified in the N terminal transactivation domain and S211 is targeted by CDK and p38 kinases whereas S226 is phosphorylated by JNK pathway. Phosphorylation of the receptor modulates its transcriptional activity, alters its protein stability and subcellular location [11-14]. GR phosphorylation appears to be cell cycle dependent [15,16] and has been shown recently to be clinically relevant [17]. The conclusions from several studies indicate that UV activated JNK and p38 MAPKs affect GR transcriptional activity and specificity in a cell type and target gene dependent manner [10,13] and hence resistance to GCs dependent apoptosis might derive from aberrant changes in these signalling pathways.

The current concept for GR-dependent apoptosis in leukaemia entails the presence of a transcriptionally competent GR [18,19] and accumulating evidence suggests that dexamethasone-induced apoptosis in lymphocytes is executed through the intrinsic pathway [3,6,8,20,21]. In agreement with these observations, knockouts of various Bcl-2 family members such as Bim [22], Puma or NOXA [23], or double knockouts of Bax and Bak confer resistance to GC mediated apoptosis in thymocytes [24]. Furthermore, microarray analysis has revealed that several pro-apoptotic members of the Bcl-2 family, such as the BH3-only molecules BMF, Bim and NOXA are induced, whereas anti-apoptotic members of this family are repressed in a glucocorticoid dependent manner [25-28]. The molecular mechanisms by which GR regulates apoptosis in a cell-type specific manner have been a subject of intense research and recently the important role of the balance of the Bcl-2 family genes determining the outcome of the GC dependent apoptotic events has been suggested [27].

Mutations or alterations in GR protein levels are uncommon in primary leukaemia cells from GC-resistant patients [29] therefore suggesting that signalling pathways are likely to play a role in modulating GR phosphorylation and activity and in determining resistance or sensitivity to GCs induced apoptosis. In addition, phosphorylation affecting the interaction and subcellular localisation of the Bcl-2 family members eventually leading to the blockade of apoptosis and hence resistance to glucocorticoids in leukaemia has been proposed as possible mechanism favouring antiapoptotic state in leukaemic cells [30].

Knockdown of the anti-apoptotic Bcl-2 family member Myeloid Cell Leukaemia sequence 1 (Mcl-1) has been shown to sensitise Acute Lymphoblastic Leukaemia (ALL) cell lines to GC-induced apoptosis [31] and is also implicated in resistance to GC-induced apoptosis in human neutrophils [32]. A critical role for the Mcl-1 function appears to be its interaction with other Bcl-2 family members and the pro-apoptotic Bcl-2 family member NOXA is essential in triggering Mcl-1 degradation [33].

In this study, we have investigated the role of glucocorticoids in the regulation of NOXA and Mcl-1 function in epithelial or lymphoid cell lines and we identified GR transcriptional involvement in the expression of both NOXA and Mcl-1. In addition, we provide evidence that NOXA and Mcl-1 expression is selectively regulated in cell types that are sensitive or resistant to glucocorticoid-induced apoptosis. Furthermore, our results demonstrate that JNK pathway activated by UV radiation alters glucocorticoid dependent transcriptional regulation of Mcl-1, Noxa and Bim and modulates GR phosphorylation pattern as well as cell cycle progression and apoptosis suggesting that these events could be important factors determining sensitivity or resistance to GC-induced apoptosis.

## Results

### The regulatory regions of the promoters of Mcl-1 and NOXA genes bear functional GREs

**Figure 1:**
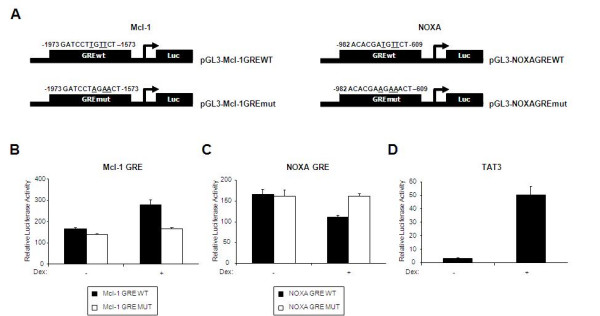
**NOXA and Mcl-1 bear functional GREs**. A, Schematic representation describing construction of luciferase reporter plasmids containing wt and mutant human NOXA and Mcl-1 GRE promoter fragments. The numbers above the constructs indicate the region of the promoter relative to the transcription start site. Mutated bases are underlined. A549 cells were either transfected with NOXA-GREwt or NOXA-GREmut (B), Mcl-1-GREwt or Mcl-1-GREmut (C) and TAT3 (D) luciferase reporter constructs. Cells were treated with Dex (100 nM) for 16 h and luciferase assays were performed [53]. The graphs represent relative luciferase to β-galactosidase activity and data is the average of 5 independent experiments.

The regulation of the balance of anti-apoptotic and pro-apoptotic members of the Bcl-2 family determines the cellular fate in the glucocorticoid mediated apoptosis [27]. Mcl-1 and Noxa have been proposed as major regulators of the glucocorticoid mediated pro- or anti- apoptotic events [34]. Therefore, we investigated whether GR was involved in the transcriptional regulation of the expression of those two genes. Towards this direction, we searched for the possible existence of GREs in the promoters of NOXA and Mcl-1 genes using the consensus GRE sequence described by Wang at al., 2004 [35]. Putative GREs were identified within the promoters of both genes. To test whether these GREs were functional, we generated luciferase reporter constructs using a ~400 bp DNA fragment from the promoters of Mcl-1 (-1973 to -1573) and NOXA (-982 to -609) containing wild type GREs or their mutated counterparts, which we constructed as described in the Figure 1A. Luciferase reporter assays were carried out in A549 human lung cancer cells. Hormone induced ~1.5-fold increase in the luciferase expression driven by the wild type Mcl-1 promoter, whereas in the case of the NOXA wild type reporter there was ~1.5 fold reduction of luciferase expression. Mutation of the NOXA and Mcl-1 GREs rendered the constructs unresponsive to dexamethasone treatment (Figure 1B and 1C). The extensively characterised GR transcription target TAT-luciferase reporter was used as control in these experiments (Figure 1D).

### NOXA and Mcl-1 are differentially regulated by glucocorticoids

**Figure 2:**
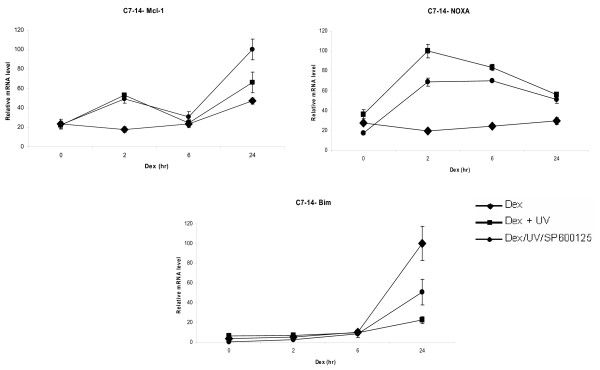
**Dexamethasone regulates NOXA/Mcl-1 mRNA levels in CEM-C7-14 cells**. CEM-C7-14 cell were cultured in DCC-treated media and incubated with either dexamethasone (1 μM) alone or combination of dexamethasone (1 μM) with UV irradiation or SP600125 inhibitor as described in Material and Methods and [10] for the times indicated. Cells were lysed and total mRNA was extracted as described in Materials and Methods, reverse transcribed and subjected to qRT-PCR for Mcl-1, NOXA and Bim. All results have been normalised to Rpl19, which was used as an internal control. The data shown is representative of three independent experiments.

**Figure 3:**
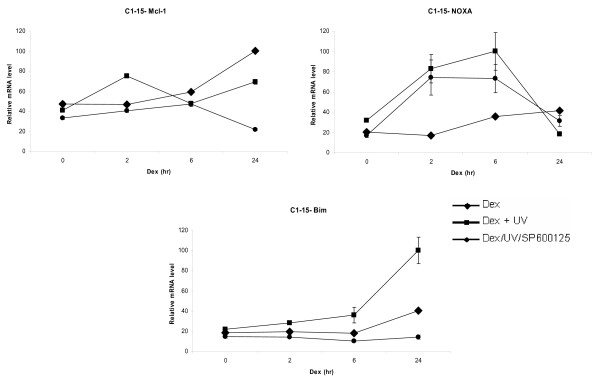
**Dexamethasone regulates NOXA/Mcl-1 mRNA levels in CEM-C1-15 cells**. CEM-C1-15 cells were cultured in DCC-treated media and incubated as described above for CEM-C7-14 cells. Cells were lysed and total mRNA was extracted as described above and subjected to qRT-PCR for Mcl-1, NOXA and Bim. All results have been normalised to Rpl19, which was used as an internal control. The data shown is representative of three independent experiments.

**Figure 4:**
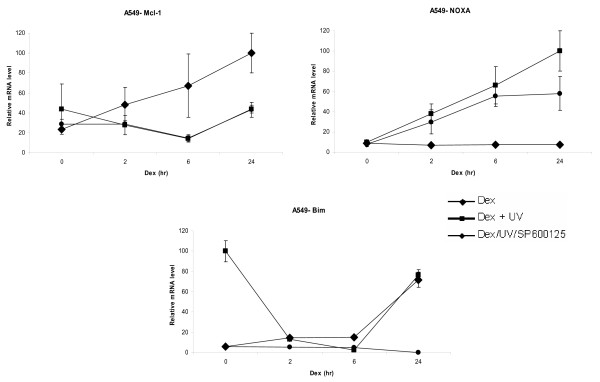
**Dexamethasone regulates NOXA/Mcl-1 mRNA levels in A549 cells**. A549 cells were cultured in DCC-treated media and incubated as described in Figure 2 above for CEM-C7-14 cells. Cells were lysed and total mRNA was extracted as described in above and subjected to qRT-PCR for Mcl-1, NOXA and Bim. All results have been normalised to Rpl19, which was used as an internal control. The data shown is representative of three independent experiments.

In order to analyse the cellular effects of the GR mediated transcriptional regulation of Mcl-1 and NOXA, we exploited the ALL cell lines CEM-C7-14 and CEM-C1-15 that are sensitive or resistant to the GC mediated apoptosis respectively. For this purpose, we examined the effects of glucocorticoids on the Mcl-1, NOXA and Bim mRNA levels, in CEM-C7-14 and CEM-C1-15 cells (Figures 2 and 3). Given the fact that phosphorylation of glucocorticoid receptor modulates its multiple functions in a target gene specific manner [10,12,13,15,16] we investigated whether UV dependent phosphorylation of GR resulted in selective modulation of Mcl-1, NOXA or Bim gene expression. For this purpose, UV irradiation was used to activate JNK mediated phosphorylation of GR and the effects of this activation on the Mcl-1, NOXA and Bim gene expression were analysed by qRT-PCR (Figures 2, 3, 4). The effects of glucocorticoid receptor activation on endogenous Mcl-1, NOXA and Bim genes were analysed in cells treated with the synthetic glucocorticoid dexamethasone for 2, 6 and 24 hours. Dexamethasone treatment of CEM-C7-14 cells resulted in a two-fold increase of Mcl-1 mRNA levels (Figure 2, diamonds). Surprisingly, combinatorial treatment of these cells with dexamethasone and with either UV or JNK inhibitor SP600125 produced similar stimulatory effect on the Mcl-1 mRNA expression in the first 6 hrs whereas JNK inhibitor further activated Mcl-1 gene expression at 24 hr of treatment (Figure 2, compare squares to circles respectively). NOXA gene expression was marginally reduced by dexamethasone treatment alone whereas MAPK dependent phosphorylation increased NOXA gene expression in CEM-C7-14 cell line (Figure 2, compare diamonds to squares). The addition of SP600125 kinase inhibitor to the UV treated CEM-C7-14 cells reduced the mRNA levels of this pro-apoptotic gene compared to the UV treatment alone at shorter treatments (Figure 2, compare squares to circles). Finally, Bim mRNA levels increased 10 fold 24 h after dexamethasone treatment of CEM-C7-14 (Figure 2, lower panel, diamonds). UV treatment had negative effect on Bim mRNA expression and this downregulation was partially reversed by treating the sensitive CEM-C7-14 cells with the kinase inhibitor (Figure 2, lower panel, compare squares to circles respectively).

The results shown in Figure 3 indicated two fold increase of Mcl-1 mRNA levels after dexamethasone treatment whereas combination of dexamethasone and UV treatments led to initial increase after two hours and significant further reduction in CEM-C1-15 cells (Figure 3, compare diamonds with squares). The use of SP600125 inhibitor revealed that Mcl-1 mRNA levels in UV treated cells were regulated by JNK mediated phosphorylation in a complex manner (Figure 3, circles). Hormone treatment increased NOXA mRNA levels after 6 and 24 h. Interestingly, when these cells were treated with UV, NOXA mRNA levels increased three fold at 6 h and dropped 24 h after dexamethasone addition to lower levels than those in dexamethasone alone treated cells (Figure 3, compare diamonds to squares at 24 h). The SP600125 inhibitor partially reversed the UV effect and NOXA mRNA levels under these conditions were close to basal levels (Figure 3, compare circles to squares), indicating that phosphorylation is important for events mediating NOXA gene expression and that JNK pathway was playing a role in this process in CEM-C1-15 cells. Significant increase in Bim mRNA levels was observed 24 h after hormone addition in CEM-C1-15 cells exposed to UV irradiation and this effect was completely abolished by SP600125 (Figure 3, lower panel, compare squares to circles respectively) signifying that JNK phosphorylation was important in the regulation of Bim gene expression.

To address tissue specific effects of glucocorticoid receptor activation, endogenous Mcl-1 and NOXA genes were analysed by monitoring their mRNA levels in A549 cells treated as above (Figure 4). Five-fold increase in Mcl-1 expression after 24 hr treatment of A549 cells with hormone was detected (Figure 4, diamonds) whereas NOXA gene expression was weakly repressed (Figure 4, diamonds). As a control treatment, we followed the Bim gene expression, as this gene is known to be an indirect glucocorticoid receptor target inducing apoptosis [28]. Bim expression increased substantially in the 24 h ligand treated cells (Figure 4, lower panel, diamonds). UV inhibited Mcl-1 expression and activated NOXA (Figure 4, squares). Bim gene expression was reduced in cells treated with dexamethasone for 6 h in combination with UV irradiation and was elevated in UV treated cells incubated in the absence of hormone (Figure 4, squares). Inhibition of JNK kinase activity by SP600125 had marginal effect on the Mcl-1 and NOXA gene expression in A549 cells, whereas it completely abolished Bim gene expression (Figure 4, circles). Taken together the results shown in Figure 4 imply that GR is involved in the transcriptional modulation of Mcl-1 and NOXA genes in A549 cells and that this regulation is UV sensitive.

In conclusion, Bim in CEM-C1-15 was activated and in CEM-C7-14 cells was inhibited by UV dependent phosphorylation and this effect was mediated at least in part by GR and JNK pathways. Bim expression was sensitive to dexamethasone and JNK inhibitor treatments. Mcl-1 gene expression increased at 2 hrs after Dexamethasone and UV and decreased after longer treatments in CEM-C1-15 cells (Figure 3), whereas UV activated hormone dependent effects on Mcl-1 gene expression in CEM-C7-14 cells (Figure 2). The effect of UV on NOXA gene expression differed between CEM-C7-14 and CEM-C1-15 cells since the 24 hrs treatments in the first case increased and in the second decreased NOXA gene expression displaying differential sensitivity to JNK mediated events in the two cell lines. Taken together the results presented in Figures 2, 3 and 4 suggested that GR dependent transcriptional regulation of Mcl-1 and NOXA gene expression is cell type specific and that the magnitude and the direction of this control is sensitive to UV radiation and JNK activation. These effects are most likely result of direct transcriptional regulation by GR since mRNA levels for both Mcl-1 and NOXA genes did not decrease substantially up to 6 hrs of treatment with dexamethasone in the absence or presence of cyclohexamide (Additional file 1).

**Figure 5:**
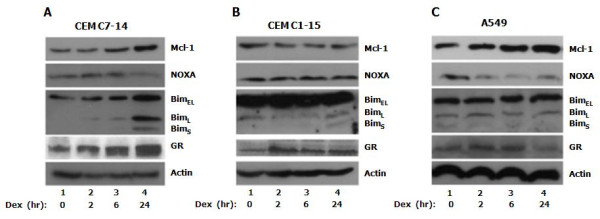
**Dexamethasone regulates NOXA/Mcl-1 protein levels**. CEM-C7-14 (A), CEM-C1-15 (B) and A549 (C) cells were cultured in DCC-treated media and incubated with dexamethasone (1 μM) for the times indicated. Cells were lysed and total cell protein extracts were subjected to western blot analysis. Mcl-1, NOXA, Bim and GR (2F8 antibody) were detected using specific antibodies against these proteins. Actin was used as loading control.

In order to substantiate these results we monitored the protein levels of the three Bcl-2 family members Mcl-1, NOXA and Bim in CEM-C7-14, CEM-C1-15 and A549 cells (Figure 5). In CEM-C7-14 cells, GR, Mcl-1 and Bim protein levels were upregulated 24 hrs after hormone treatment (Figure 5A, lane 4). Initially increased NOXA protein levels following 2 hrs hormone treatment were observed (Figure 5A, NOXA, lane 2) declining later, 24 hrs after the hormone addition (Figure 5A, NOXA, compare lane 2 to 4) in agreement with previously published results [27,28]. In CEM-C1-15 cells, Mcl-1, NOXA, and Bim protein levels remained relatively unchanged irrespectively of the duration of the hormone treatment whereas GR protein levels increased after 2 hours of hormone treatment (Figure 5B, lanes 1-4). In A549 cells Mcl-1 protein levels were upregulated (Figure 5C, Mcl-1) whereas NOXA protein levels were downregulated after 24 h treatment with dexamethasone (Figure 5C, NOXA, lane 4). Bim protein levels did not change and GR was weakly downregulated at 24 hours of treatment after initial increase at shorter treatments (Figure 5C, GR lanes 1-4). To conclude, Mcl-1 protein levels increased in CEM-C7-14 and A549 cells and were unchanged in CEM-C1-15 cells. NOXA protein levels decreased in CEM-C7-14 and A549 whereas remained unaltered in CEM-C1-15 cells. Bim protein levels increased only in CEM-C7-14 cells.

### Glucocorticoid receptor is differentially phosphorylated in UV irradiated CEM-C7-14, CEM-C1-15 and A549 cells

**Figure 6:**
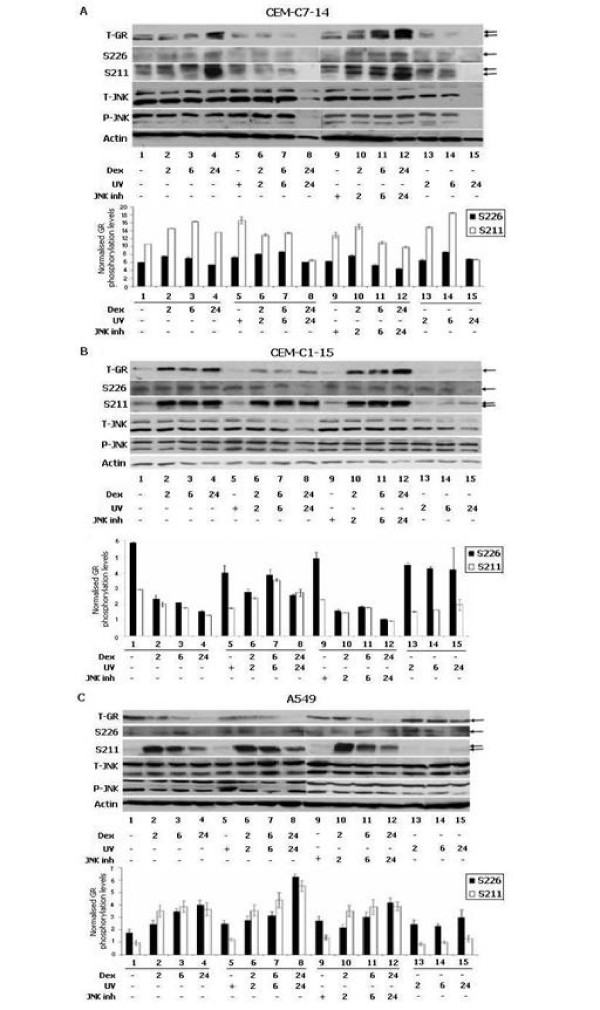
**JNK or CDK mediated GR phosphorylation occurs in a cell type dependent manner**. CEM-C7-14 (A), CEM-C1-15 (B) and A549 (C) cells were cultured in DCC-treated media and incubated with dexamethasone (1 μM) (lanes 1-12), UV (lanes 5-8 and 13-15) and the SP600125 inhibitor (lanes 9-12) for the times indicated. Cells were treated with UV or JNK inhibitor and collected after 30 min in lanes 5 and 9, or after indicated time. Cells were lysed and total cell protein extracts were subjected to western blot analysis using the specific antibody 2F8 against total GR (T-GR), phosphorylated GR at S226 (S226) and S211 (S211), total JNK (T-JNK, JNK2/3 54 kDa and JNK1 46 kDa) and phosphorylated JNK (P-JNK). The bands indicated by arrow were taken into account for the quantification shown in the bottom of A, B and C. Actin was used as loading control.

Kinase pathways affecting GR phosphorylation have been implicated as an important factor in determining the effects of glucocorticoids [10,13]. Our results suggested that UV and JNK activation play a role in determining GR transcriptional activity (Figures 2, 3, 4, and [10]). In an attempt to analyse GR phosphorylation at S226 and S211 target sites, CEM-C7-14 cell lines were UV irradiated to activate the MAPK pathway [10] or were treated with the SP600125 inhibitor of JNK activity in the presence or absence of dexamethasone for different time intervals as shown in Figure 6A. Two GR bands were observed after probing with GR specific antibodies in CEM-C7-14 cells, perhaps due to the existence of multiple GR isoforms carrying other posttranslational modifications in addition to S211 phosphorylation, or other mechanisms. We observed that the total GR protein levels and S211 phosphorylation gradually increased with hormone treatment in the presence or absence of the SP600125 inhibitor, whereas the phosphorylation levels of GR at S226 were generally low and followed the total GR protein levels (Figure 6A, lanes 1-4 and 9-12). UV treatment alone or in combination with hormone resulted in general decrease of the total and both phosphorylated GR isoforms (Figure 6A, lanes 5-8 and 13-15 respectively). Total and phosphorylated JNK levels were used as control for the MAPK activity and actin as loading mark for equal protein amounts (Figure 6A). Comparative densitometric analysis of the GR phosphorylation levels indicated prevalence of GR phosphorylation at the S211 residue versus the S226 in the CEM-C7-14 cells (Figure 6A, lower panel). Furthermore, the possibility of the existence of more than one GR isoform in the CEM-C7-14 cells cannot be excluded as more than one band was detected in immunoblots indicated by arrows (Figure 6A, arrows). The bands specified with arrows in Figure 6A were considered for the quantification presented in Figure 6A, lower panel.

In CEM-C1-15 cells, the total GR protein levels increased two hours after hormone treatment alone or in combination with SP600125 inhibitor and remained relatively unaltered with prolonged treatments (Figure 6B, lanes 1-4 and 9-12). In cells treated with combination of hormone with UV or UV alone decrease in total GR protein levels was observed (Figure 6B, lanes 5-8 and 13-15 respectively). In contrast to CEM-C7-14 in CEM-C1-15 cells, the levels of GR phosphorylation at S226 did not follow the total GR protein levels (Figure 6B). Phosphorylation of GR at S211 increased 2 hours after the addition of hormone and did not significantly change in cells treated with hormone/SP600125 or hormone/UV (Figure 6B). In cells treated with UV in the absence of hormone basal levels of S211 GR phosphorylation were detected (Figure 6B, lanes 13-15). Total JNK protein levels and its activity measured by its phosphorylation status, together with actin loading control are displayed in Figure 6B. Densitometric scanning of these results and normalization of phosphorylation levels to the total GR levels indicated that S226 phosphorylation was the highest in the absence of hormone and revealed overall predominant or equal phosphorylation of the S226 over the S211 GR phosphorylation (Figure 6B, lower panel). The bands specified with arrows in Figure 6B were quantified and results presented in the diagram (Figure 6B, lower panel).

The fact that CEM-C1-15 cell lines are resistant and the CEM-C7-14 are sensitive to glucocorticoid induced apoptosis prompted us to investigate the phosphorylation pattern of the receptor in another cell line namely the A549 human lung epithelial cells [36]. The reason for monitoring the GR phosphorylation pattern in A549 cells was to test whether cell type influences links between GR phosphorylation and resistance or sensitivity to glucocorticoid-stimulated apoptosis (Figure 6C). The results shown in Figure 6C indicated that GR protein abundance in A549 cells decreased upon hormone treatment alone or in combination with UV or JNK inhibitor (Figure 6C, lanes 1-12). UV irradiation did not change significantly total GR protein levels (Figure 6C, lanes 5-8 and 13-15). S226 phosphorylation increased upon 2 and 6 hours of hormone treatment and slightly decreased after 24 hrs of Dex treatment (Figure 6C, lanes 1-4). Significant increase in S226 phosphorylation was observed in cells treated with UV alone compared to non treated cells (Figure 6C, lower panel, compare black bar 1 with bar 5) and after 24 hrs treatment with combination of UV and hormone compared to individual treatments (Figure 6C, lower panel, compare black bar 1 with black bar 5 and black bars 4 and 15 with black bar 8). S211 phosphorylation gradually decreased after initial increase in cells treated with hormone for 2 hrs (Figure 6C, lanes 1-12). In UV irradiated A549 cells, phosphorylation of GR at S211 remained low at the basal level (Figure 6C, lanes 13-15). The bands specified with arrows in Figure 6C were quantified and results presented in the diagram (Figure 6C, lower panel). Quantification of these results suggested that in most cases S211 and S226 residues were phosphorylated to similar extent, except in the UV treated cells where S226 phosphorylation was more intense than S211 (Figure 6C, lower panel, compare black to white bars). Taken together these results indicated a differential role of the GR phosphorylation in the three cell lines as detected by altered phosphorylation and kinetic patterns of CDK and JNK dependent GR target residues.

### Glucocorticoid mediated apoptosis is cell type specific

**Figure 7:**
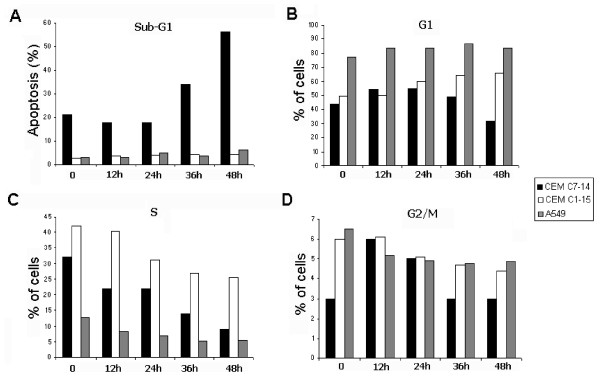
**CEM-C7-14 cells accumulate in Sub-G1 phase after prolonged hormone treatment**. CEM-C1-15, CEM-C7-14 and A549 cells (white, black and grey bars respectively) cultured in DCC-treated media incubated with Dex (1 μM) for the times indicated and FACS analysis performed as described previously [53]. Sub-G1 (A), G1 (B), S (C) and G2 (D) cell cycle phase profiles are representative of three independent experiments.

To determine the apoptotic effect that glucocorticoids have on various cell lines, we treated CEM-C7-14, CEM-C1-15, A549, and HeLa cell lines with dexamethasone and performed flow cytometry (Figure 7 and data for HeLa not shown). The lymphoblastic leukaemia CEM-C7-14 cells are sensitive to glucocorticoid-induced apoptosis, whereas the CEM-C1-15 cell line is a selected *in vitro *subclone of the CEM-C7-14 resistant to GC mediated apoptosis [37]. Although A549 lung carcinoma cells have been extensively studied with respect to GR, inconclusive results have been reported in terms of their response to glucocorticoid induced apoptosis, although previous reports have shown that ectopic GR expression induces apoptosis in these cells [36]. In CEM-C1-15 and A549 the percentage of cells in the sub-G1 phase was mostly below 10%, and a slight decrease in S phase of the cell cycle 48 h after dexamethasone treatment was observed (Figure 7A and 7C). In the case of CEM-C7-14 cells, prolonged hormone treatment resulted in the increase of the apoptotic population and concomitant decrease in the number of cells entering the S-phase of the cell cycle (Figure 7A, C and Additional file 2).

**Figure 8:**
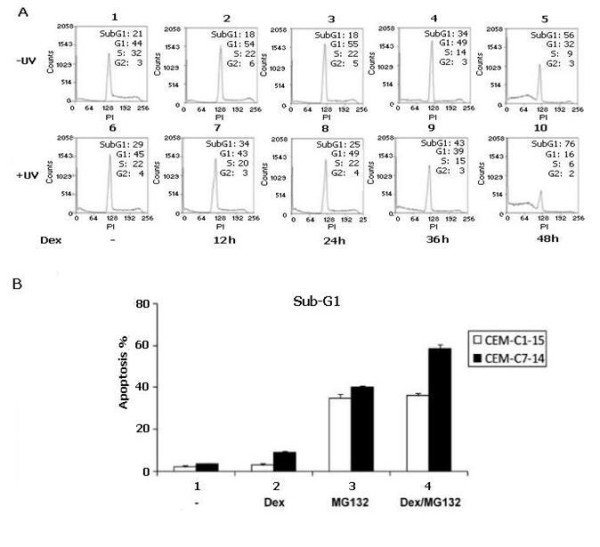
**A. UV irradiation enhances the proapoptotic effect of dexamethasone in CEM-C7-14 cells**. Determination of the cell cycle profiles of CEM-C7-14 cells UV irradiated (6-10) or not irradiated (1-5) in the presence (2-5 and 7-10) or absence (1 and 6) of dexamethasone for the indicated times. FACS experiments were performed two times and the profiles shown are from one experiment. **B. MG132 enhances glucocorticoid-induced apoptosis**. CEM-C1-15 (white bars) and CEM-C7-14 cells (black bars) were cultured in DCC-treated media incubated with dexamethasone (1 μM for 48 hr) (bars 2 and 4) in the presence of MG-132 (1 μM, 24 hr) (bars 3 and 4) and FACS analysis performed. Data is representative of three independent experiments.

The effect of phosphorylation on the apoptotic profile of CEM-C7-14 cells was investigated by treating UV irradiated or not irradiated cells with dexamethasone for the indicated times and subjecting them to FACS analysis. As shown in Figure 8A the longer treatment with dexamethasone increased the Sub-G1 population from 21% in 0 h to 56% in 48 h dexamethasone treated cells. Further increase of the apoptotic population of CEM-C7-14 cells was observed with combination of UV irradiation and hormone treatment for the time points tested. In particular, only a quarter of the UV irradiated CEM-C7-14 cells was alive after 48 h of dexamethasone treatment, whereas half of dexamethasone only treated cells was still alive after 48 h treatment (Figure 8A, compare profile 5 with 10).

Since NOXA mRNA expression was differentially regulated in UV treated CEM-C7-14 and CEM-C1-15 cells (Figures 2 and 3) we decided to investigate whether the expression of NOXA was important for the glucocorticoid-induced apoptosis. For this purpose, we used the proteasome inhibitor, MG-132, which has been shown to significantly induce NOXA [38] to treat CEM-C1-15 and CEM-C7-14 cells in the presence or absence of dexamethasone and determined the percentage of cells that had undergone apoptosis using flow cytometry (Figure 8B). As expected hormone treatment alone did not have any effect on apoptosis in CEM-C1-15 cells (Figure 8B compare white bars 1 and 2). Addition of MG-132 increased dramatically the Sub-G1 population in both cell lines (Figure 8B, compare bars 1 and 3), whereas combination of dexamethasone with MG-132 increased only the number of CEM-C7-14 cells undergoing apoptosis (Figure 8B, compare bars 3 and 4). In particular dexamethasone treatment increased by threefold the Sub-G1 population of CEM-C7-14 cells (Figure 8B, compare black bars 1 and 2). Combination of dexamethasone with MG-132 increased further the net apoptotic effect of MG-132 by 20% only in the CEM-C7-14 cells (Figure 8B, compare black bars 3 and 4) implying that NOXA contributes to the GC induced apoptosis only in CEM-C7-14 cells (Additional file 3).

## Discussion

GR transcriptional activity is regulated by its ligands, its interaction with cofactors and posttranslational modifications [7]. The crosstalk between diverse signalling pathways results in the activation of several kinases that phosphorylate GR [10,13,16]. The CDK family of kinases targets GR for phosphorylation at S203 and S211 whereas the JNK pathway targets S226 [10,13,39]. However, other kinases including p38 might also be involved in targeting GR directly or indirectly in a cell type specific manner [13,17]. One of the most puzzling properties of this group of steroid hormones is their role in programmed cell death. Dexamethasone stimulates apoptosis in a variety of cells of the immune system and it is used extensively as a therapeutic agent in leukaemia, while in most other cell types it exerts anti-apoptotic or no effects [2]. Several mechanisms have been proposed to explain the cell type specificity of the apoptotic effects of glucorticoids including versatile expression of GR isoforms in different cell types [40] alternative subcellular localisation [41] decreased proteasomal activity in hormone treated cells [42], and posttranslational modifications [10,13].

The GR dependent transcriptional regulation of Bcl-2 family members has been proposed as one mechanism of mediation of the opposing apoptotic effects of dexamethasone in different cell types [20,21,27]. The GR inducible target Bcl-x gene for example exhibits tissue specific pattern of promoter usage explaining the distinction between the pro- and anti-apoptotic effects of glucocorticoids in lymphoid versus non lymphoid cells [43]. Recent analysis has identified several other members of the Bcl-2 family to be targeted by GR including Mcl-1 and NOXA and the determination of apoptosis or survival outcome has been attributed to the balance between pro- and anti-apoptotic genes of the Bcl-2 family [21,44].

In this study we investigated the molecular mechanisms underlying the transcriptional effects of glucocorticoids and the signalling pathways controlling the Bcl-2 family members. Of particular interest were the NOXA, Mcl-1 and Bim members of the Bcl-2 family since they have been implicated in the apoptotic regulation of various forms of leukaemia [28,31,45,46]. We identified putative GREs in the promoter regions of Mcl-1 and NOXA (Figure 1A) and assessed their functionality using luciferase reporter assays (Figure 1B and 1C). The changes in luciferase expression driven by the Mcl-1 or NOXA promoter were mediated by GR since mutated GREs were unresponsive to hormone treatment (Figure 1B and 1C). Direct GR regulation of other Bcl-2 family members at the transcriptional level and the role of these genes in glucocorticoid-induced apoptosis have been shown in other reports [18,25-27,44].

To monitor the hormone dependent effects on the expression of Mcl-1, NOXA and Bim mRNA levels we employed qRT-PCR analysis (Figures 2, 3, 4). Twofold induction of Mcl-1 mRNA was observed in CEM-C7-14 and CEM-C1-15 cells and five fold in A549 cells. Bim gene expression increased substantially in A549 and CEM-C7-14 cells treated by glucocorticoids whereas only two fold induction of this gene was evident in CEM-C1-15 cell lines. Noxa gene expression was weakly inhibited by glucocorticoids in CEM-C7-14 and A549 cells whereas glucocorticoid dependent increase was observed in CEM-C1-15 cells. Combination of 24 h dexamethasone and UV treatment inhibited Mcl-1 in CEM-C1-15 and induced this gene in CEM-C7-14 cells compared to hormone treatment alone in a JNK dependent manner (Figures 2 and 3, compare diamonds to squares at 24 h time point). NOXA was induced by UV treatment in A549 cells at all time points tested and in CEM-C1-15 cells treated with hormone for 2 and 6 hrs, whereas repression of this gene was observed in cells treated with hormone for 24 h when compared to those cells that were treated with dexamethasone alone (Figures 3 and 4, compare diamons to squares). In contrast, under the same conditions two times elevated mRNA NOXA levels were observed in CEM-C7-14 cells at 24 hrs of treatment (Figure 2, compare diamonds to squares at the 24 h time point). In UV irradiated and dexamethasone treated for 24 h CEM-C1-15 cells Bim mRNA levels were increased whereas the opposite was observed in CEM-C7-14 cells where the Bim mRNA levels were dramatically decreased under the same conditions (Figures 2 and 3, compare diamonds to squares at the 24 h time point). The kinase inhibitor completely abolished the effect of UV on Bim levels in CEM-C1-15 cells and partially abolished that effect in CEM-C7-14 cells (Figures 2 and 3, circles at the 24 h time point). Our results are in agreement with previously reported data indicating that glucocorticoids have opposing effects even on the same gene depending on the cell type as well as distinguishing characteristics of the signalling pathways [43]. Similar results in regards to NOXA gene expression being subject to differential regulation by different chemotherapeutic agents have recently been reported [45]. Furthermore, these data link the effects mediated by glucocorticoids on Bcl-2 family members' gene expression to the activation of the JNK pathway.

Given the fact that Mcl-1 has a relatively short half-life being targeted by NOXA for degradation [33] and has been implicated in the resistance to GC-mediated apoptosis [31,32] we next tested the effects of dexamethasone treatment on their protein levels (Figure 5). We observed no changes of Mcl-1, NOXA or Bim protein levels in CEM-C1-15 cells irrespectively of the duration of the hormone treatment (Figure 5B). In CEM-C7-14 cells the protein levels correlated with the mRNA levels (Figure 2, diamonds, and Figure 5A). These results suggest that the regulation of NOXA/Mcl-1 gene expression by glucocorticoids and potentially of Mcl-1 stability could be a factor determining protection against hormone induced programmed cell death in CEM-C1-15 and sensitivity in CEM-C7-14 cells (Figure 5 and data not shown).

Accumulating evidence suggests potential crosstalk between the UV irradiation and glucocorticoids in controlling the programmed cell death [47]. We have recently reported that in UV irradiated cells GR is phosphorylated in a JNK dependent manner at S226 [10]. We and other research groups have reported that elevation of S226 phosphorylation of GR results in the reduction of the CDK dependent S211 phoshorylation [13,39]. S226 phosphorylation is originally thought to impose negative whereas S211 stimulating effect on GR transcriptional activity, although target gene specificity of these phosphorylations is emerging as a new concept [10,13,39]. To investigate any possible link between Mcl-1 and/or NOXA mRNA expression and predominance of S226 or S211 phosphorylated GR isoforms we followed the GR phosphorylation status in all three cell lines treated with UV as shown in Figure 6. Predominant GR phosphorylation at S211 was observed in CEM-C7-14 cells compared to S226 phosphorylation levels normalised to total GR protein levels. On the contrary, in CEM-C1-15 cells the S226 phosphorylation levels were prevailing to those of S211 indicating potential differential MAPK/CDK pathway activity in the two cell lines. Another explanation for the distinctive hormone effects in CEM-C7-14 and CEM-C1-15 cell lines could be the existence of diverse cell line specific GR isoforms (Figures 6A and 6B respectively). Interestingly in A549 cells S211 and S246 residues displayed mostly similar phosphorylation patterns. In A549 and CEM-C1-15 cells UV irradiation in the absence of hormone shifted the balance towards S226 phosphorylation (Figures 6B and 6C).

Taken together these results indicate that phosphorylation of GR predominantly at S211 or S226 is a result of the activation of at least two distinct signalling pathways in CEM-C1-15 and CEM-C7-14 cell lines that are disproportionately targeting GR for phosphorylation in these two cell lines. It has been suggested that phosphorylation plays a crucial role in the regulation of GR protein stability since mutation of all GR phosphorylation sites abolished the receptor's hormone dependent degradation [11,48]. GR protein stability on the other hand is a crucial factor in determining its transcriptional activity [11]. It is possible that deregulation of both the receptor's protein stability and transcriptional activity by MAPK and CDK pathways contributes to the sensitive versus resistant to GCs induced apoptosis phenotype in different cell lines. Upregulation of GR protein levels has been detected after short and long term dexamethasone treatments in CEM-C1-15 cells (Figure 6B). A possible explanation for the inability of the accumulated GR to induce apoptosis in CEM-C1-15 cells (Figure 6B) is that GR phosphorylation at S226 increases the GR protein stability but renders it transcriptionally inactive [10] and data not shown. This possibility is currently under investigation in our laboratory.

Consistent with previously published observations, sub-G1 apoptotic cells were mostly detected in CEM-C7-14 and not in CEM-C1-15, A549 and HeLa cells (Figures 7 and 8 and data not shown) [49]. The significance of the enhanced NOXA expression in the glucocorticoid and UV/hormone mediated apoptosis was confirmed in CEM-C7-14 cells treated with the proteasome inhibitor MG-132, which is a potent inducer of NOXA protein levels [38]. In agreement with previously published observations [50], the combined treatment of dexamethasone and MG132 resulted in increased percentage of apoptotic CEM-C7-14 cells in comparison to cells treated with MG-132 alone (Figure 8, compare black bars 3 and 4). Treatment of CEM-C1-15 cells with dexamethasone and MG132 did not change the level of MG-132 induced apoptosis in these cells (Figure 8, compare white bars 3 and 4).

## Conclusions

In conclusion, this report describes the complex cell type specific molecular mechanisms through which glucocorticoid mediated transcription and UV induced signalling regulate the NOXA/Mcl-1 balance and determine resistance versus sensitivity to glucocorticoid induced apoptosis. Given the fact that glucocorticoids are used extensively in the treatment of ALL and to prevent sickness during chemotherapy the results described here could be used towards improving glucocorticoid based therapies [51].

## Materials and methods

### Cell culture and antibodies

Cells were grown at 37°C and 5% CO_2_. A549 cells maintained in Dulbecco's minimum essential medium (DMEM, Gibco) whereas CEM-C1-15 and CEM-C7-14 cells were cultured in RPMI medium and all media supplemented with 10% fetal bovine serum (FBS, Gibco) and 10 units/ml each of penicillin and streptomycin (Gibco). DCC treated serum (Hyclone) was used in all experiments before dexamethasone treatment. The following antibodies were used for western blotting and immunoprecipitation: Actin, Mcl-1 and Bim (Abcam), NOXA (Alexis), GR (2F8 generously provided by Dr M.N. Alexis) [52]. The antibody against S226 phosphorylated GR was purchased from Abcam and the GR phospho-S211 was purchased from Cell Signaling. UV and JNK inhibitor treatments were carried out as described in [10].

### Western blotting

Cells were seeded into 100 mm plates and maintained in DCC-FBS media. Dex (100 nM for adherent and 1 μM for CEM cells, Sigma) was added at different timepoints. To isolate protein, cells were washed twice with ice-cold phosphate-buffered saline (PBS) and lysed in High Salt Lysis buffer (45 mM HEPES pH 7.5, 400 mM NaCl, 1 mM EDTA, 10% glycerol, 0.5% NP-40, 1 mM DTT, 1 mM PMSF, 1 μg/ml aprotinin, leupeptin and pepstatin A, 20 mM β-glycerophosphate, 5 mM sodium pyrophosphate and 2 mM sodium orthovanadate). Protein levels were measured and equal amounts of protein were loaded and resolved by SDS PAGE and Western blotting. Blots were developed with the ECL substrate according to manufacturer's instructions (Pierce). The quantification of the density of the bands on the blots was performed using Image J software and the densitometric analysis was performed three times for each band. The average of these measurements was used to calculate the density of the total GR (A) and then the density of the respective actin band (B). The same was followed for the phospho GR (C) (phospho GR S211 or phospho GR S226). The results plotted on the diagram shown in Figures 6A, B, and 6C are the products of the following formula: total GR (A)/actin (B) = X; The ratio of the intensities of total GR versus actin (X) was then used to determine the relative intensity of GR phosphoisoforms. Phospho GR (C)/(X) = relative phosphorylated GR levels (Y), which has been plotted and shown on the diagrams. The bands considered for the quantification are indicated with arrows within the relevant Figure 6.

### Quantitative RT-PCR

Adherent cells were grown to 80% confluence and CEM cells cultured to 1 × 10^7 ^cells in 30 mm well dishes. Cells were treated with Dex (100 nM for A549 and 1 μM for CEM cells) for the times indicated. CHX (30 mM) was added 1 hr prior to Dex treatment. Total mRNA was extracted using the RNeasy plus mini kit (Qiagen). RNA concentrations were measured and 1 mg/ml of RNA was reverse transcribed according to the two-step protocol (ABgene) using an oligo-dT primer (ABgene). The DNA was diluted 4-fold, which was used for qPCR analysis using SYBR^® ^Green JumpStart™ *Taq *ReadyMix™ (Sigma). Analysis was performed using Opticon monitor 3 software as described previously [53]. The primers used in this study were:

Rpl19 (F: ATGTATCACAGCCTGTACCTG, R: TTCTTGGTCTCTTCCTCCTTG),

Mcl-1 (F: TCAAAAACGAAGACGATGTGA, R:CAAAGGCACCAAAAGAAATGA),

NOXA (F: AAGAAGGCGCGCAAGAAC, R: TCCTGAGCAGAAGAGTTTGG),

Bim (F: GAGAAGGTAGACAATTGCAG, R: GACAATGTAACGTAACAGTCG).

### Luciferase reporter gene assays

To obtain DNA segments carrying GREs from the Mcl-1 and NOXA promoters, primers were designed to amplify a ~400 bp specific region (NOXA GRE (F: TGGCCTCGCCAAACATT ATGCAA, R:GAGACTTGGGTAAACAAGC CCAG) and Mcl-1 GRE (F: GAGCACTGAT GGTGCCACTGCA, R: GAAACCACATTG TCAGGCCTC). The PCR fragments were then subcloned in the Zero blunt ended TOPO vector (Invitrogen) and digested using Kpn1 and Sac1 restriction enzymes (Roche). The new PCR fragments including restriction sites were ligated into the pGL3 promoter luciferase vector (Invitrogen) and the presence of the GREs confirmed with DNA sequencing. To mutate the luciferase vectors, the QuikChange site directed mutagenesis kit was used according to manufacturers guidelines (Stratagene) and mutations confirmed with DNA sequencing. The following primers were designed for mutagenesis:

NOXA GREmutF: (CTTCCCAACTCAAACA CGAAGAACTTTCTGGCTGGCACCAGG, NOXA GREmutR: CCTGGTGCCAGCCAG AAAGTTCTTCGTGTTTGAGTTGGGAAG) and Mcl-1 GREmutF: (ATACATGGCATAT AAGAAGATCCTAGAACTCAAGGGCTTACAAACCTCTAG) and Mcl-1 GREmutR: (CTAGAGGTTTGTAAAGCCCTTGAGTTCTAGGATCTTCTTATATG CCA TGTAT). Adherent cells were plated in 30 mm dishes and transfected with indicated plasmids using Polyfect reagent (Qiagen). Cells were harvested in reporter lysis solution (Promega) and the luciferase activity was analysed using luciferin reagent following the recommended protocol (Promega) using a luminometer machine [53].

### Flow cytometry (FACS analysis)

Cells were seeded into 100 mm plates and maintained in DCC-FBS media. Dex (1 μM) was added at different time points before collection and were centrifuged at 1,200 rpm for 5 minutes (4°C). Pellets were washed with ice-cold PBS before adding 1 ml 50% EtOH/PBS drop-wise, vortexing gently. After washing with PBS, 100 μl of 125 U/ml ribonuclease A (Sigma) and 400 μl of 50 μg/ml PI (Sigma) were added. The samples were incubated at 37°C for at least 30 min before cell cycle analysis. The representations and percentages of cell cycle phases were analysed by Modfit software [53].

## Competing interests

The authors declare that they have no competing interests.

## Authors' contributions

JTL planned and performed experiments, analysed the results, prepared the draft of the manuscript and this study consists part of his PhD thesis.

RR has planned, repeated and analysed the results of the qRT-PCR experiments.

GX has planned and performed the FACs experiments with UV irradiated cells.

IB has planned, repeated and analysed the results of the Western blot experiments.

CD has formed the hypothesis of the research in collaboration with MKD, supervised the research carried out, interpreted the results and prepared the manuscript.

MKD has formed the hypothesis of the research in collaboration with CD, supervised the experiments carried out in her laboratory and contributed to the discussion and writing of the manuscript. Substantial part of the funding for this project was provided by MKD's research grant [Wellcome Trust (069024)].

All authors read and approved the final manuscript.

## Supplementary Material

Additional file 1 **Supplementary Figure 1.** Direct effects of glucocorticoids on Mcl-1, NOXA and Bim gene expression. CEM C1-15 (A), CEM C7-14 (B) and A549 (C) cells were cultured in DCC-treated media and incubated with dexamethasone alone for the times indicated (solid line) or pre-treated with cyclohexamide (30 μM) 1 hour prior to dexamethasone treatment (dashed line). RNA was extracted, reverse transcribed and used in a qRT-PCR reaction with primers to analyse the specific mRNA indicated. All results have been normalised to Rpl19 as an internal control. Graphs show the average of at least 3 independent experiments. Click here for file

Additional file 2 **Supplementary Figure 2. **Cell cycle profiles of dexamethasone treated cells. CEM-C1-15, CEM-C7-14 and A549 cells were treated with dexamethasone for the indicated times. Cells were harvested, stained with propidium iodide and their cell cycle profile was determined by FACS analysis. Click here for file

Additional file 3 **Supplementary Figure 3. **Cell cycle profiles of dexamethasone and MG132 treated cells. CEM-C7-14 cells were cultured in DCC-treated media incubated with dexamethasone (1 μM, 48 hr) and MG132 (1 μM, 24 hr) and FACS analysis performed. Cell cycle profiles are representative of three independent experiments. Click here for file
