# Supplementary figures and images for: The role of glucocorticoid receptor phosphorylation in Mcl-1 and NOXA gene expression
Source: Mol Cancer. 2010 Feb 15;9:38. doi: 10.1186/1476-4598-9-38 (PMC2834612; doi:10.1186/1476-4598-9-38)

## Slide 1
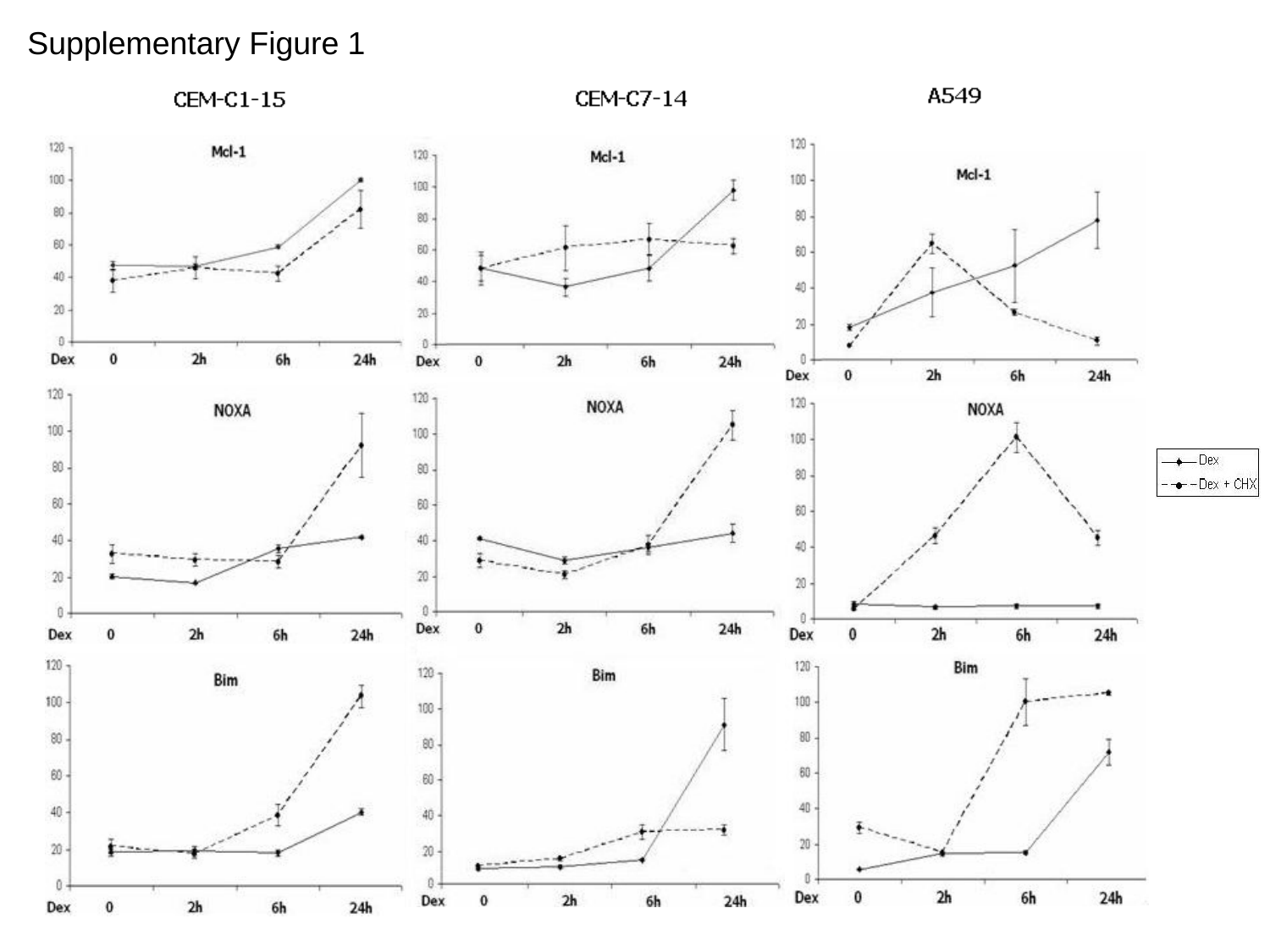

Supplementary Figure 1

Supplement: Additional file 1 — Supplementary Figure 1. Direct effects of glucocorticoids on Mcl-1, NOXA and Bim gene expression. CEM C1-15 (A), CEM C7-14 (B) and A549 (C) cells were cultured in DCC-treated media and incubated with dexamethasone alone for the times indicated (solid line) or pre-treated with cyclohexamide (30 μM) 1 hour prior to dexamethasone treatment (dashed line). RNA was extracted, reverse transcribed and used in a qRT-PCR reaction with primers to analyse the specific mRNA indicated. All results have been normalised to Rpl19 as an internal control. Graphs show the average of at least 3 independent experiments. [file 1476-4598-9-38-S1.PPT]

## Slide 1
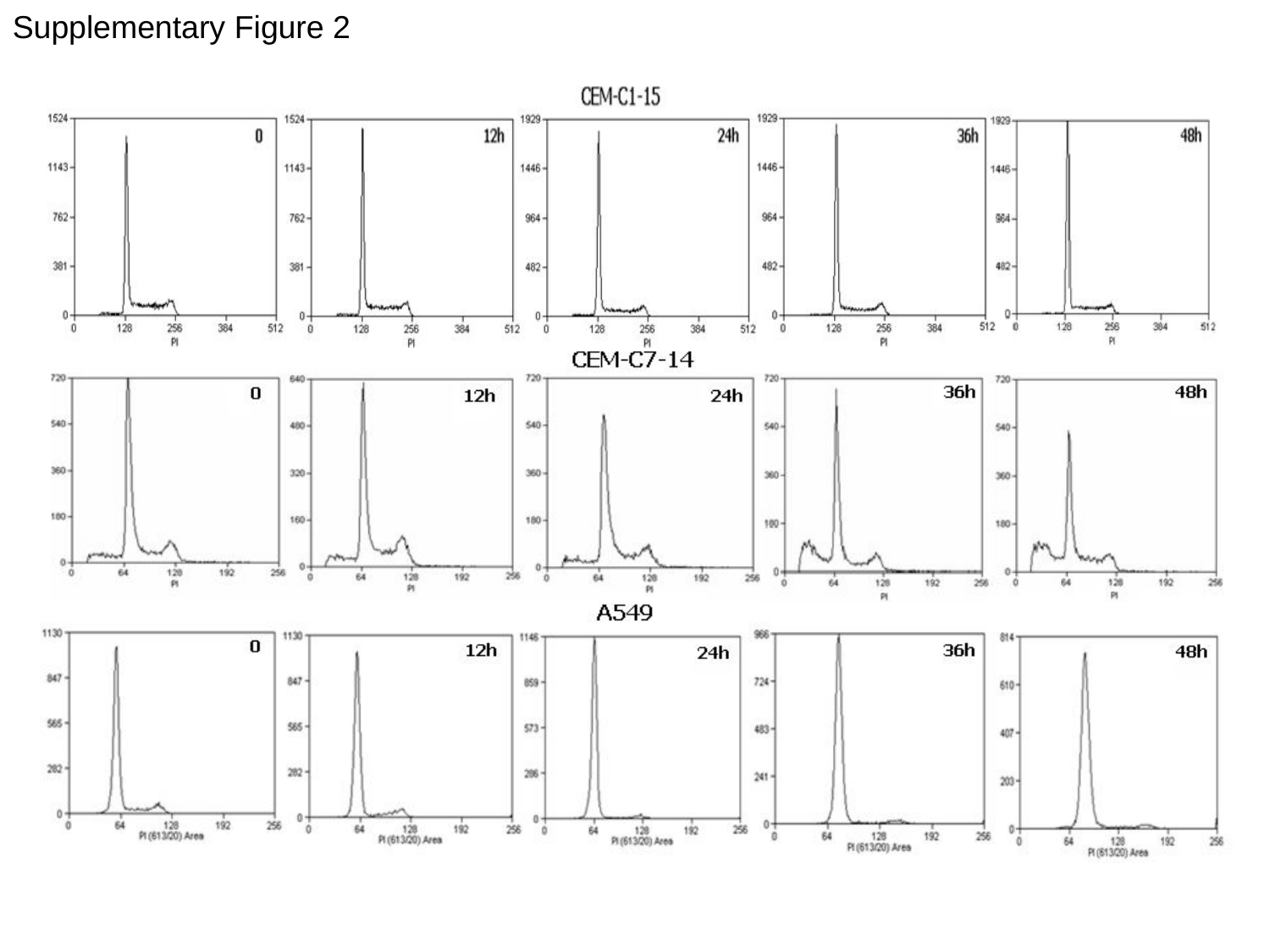

Supplementary Figure 2

Supplement: Additional file 2 — Supplementary Figure 2. Cell cycle profiles of dexamethasone treated cells. CEM-C1-15, CEM-C7-14 and A549 cells were treated with dexamethasone for the indicated times. Cells were harvested, stained with propidium iodide and their cell cycle profile was determined by FACS analysis. [file 1476-4598-9-38-S2.PPT]

## Slide 1
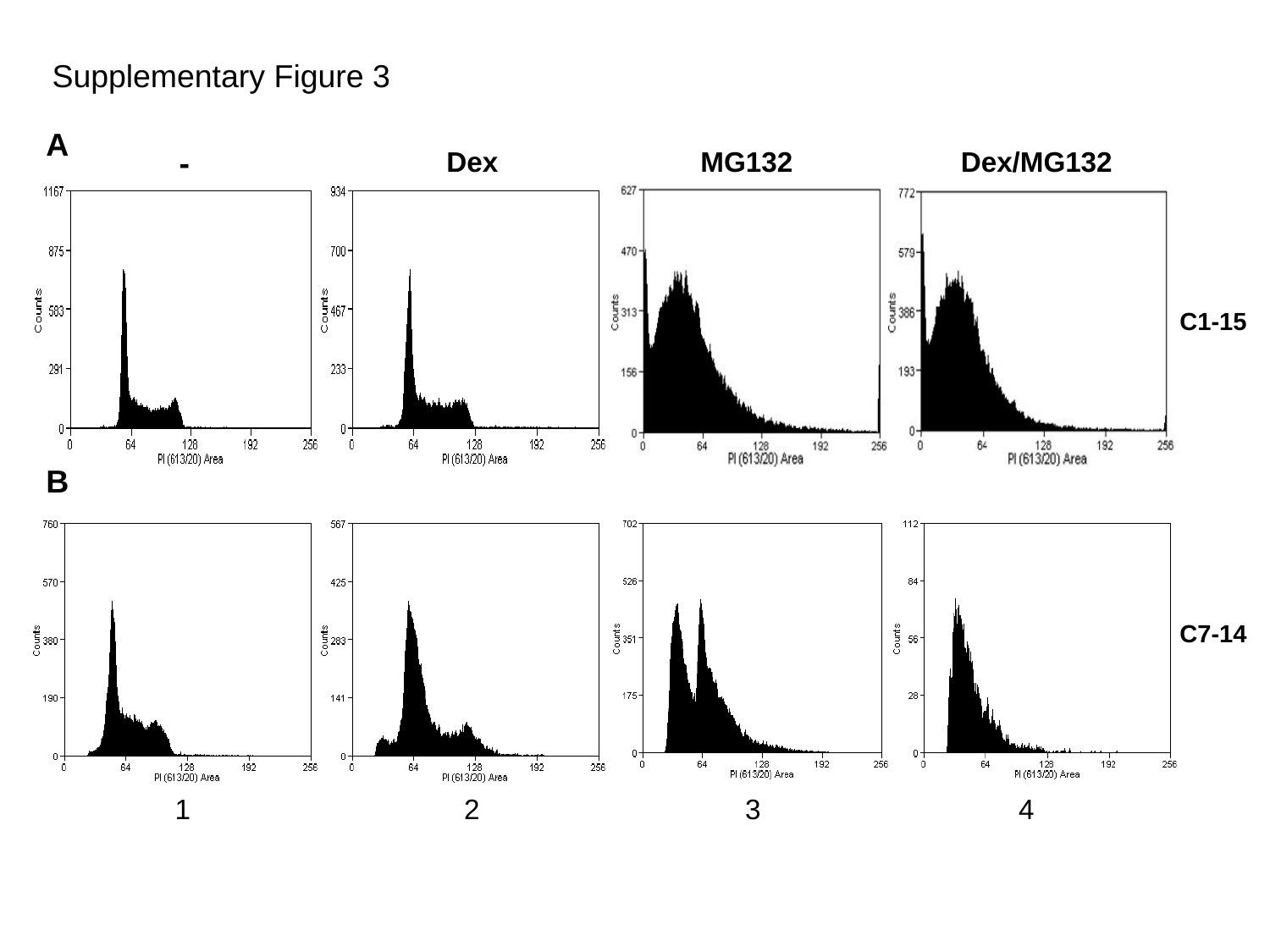

Supplementary Figure 3
A
-
Dex
MG132
Dex/MG132
C1-15
B
C7-14
1 2 3 4

Supplement: Additional file 3 — Supplementary Figure 3. Cell cycle profiles of dexamethasone and MG132 treated cells. CEM-C7-14 cells were cultured in DCC-treated media incubated with dexamethasone (1 μM, 48 hr) and MG132 (1 μM, 24 hr) and FACS analysis performed. Cell cycle profiles are representative of three independent experiments. [file 1476-4598-9-38-S3.PPT]
